# Anti-inflammatory and hepatoprotective effects of exopolysaccharides isolated from *Pleurotus geesteranus* on alcohol-induced liver injury

**DOI:** 10.1038/s41598-018-28785-0

**Published:** 2018-07-12

**Authors:** Xinling Song, Zhonghai Liu, Jianjun Zhang, Qihang Yang, Zhenzhen Ren, Chen Zhang, Min Liu, Zheng Gao, Huajie Zhao, Le Jia

**Affiliations:** 1 College of Life Science, Shandong Agricultural University, Taian, 271018 P. R. China; 2 College of Chemistry, Nanjing Tech University, Nanjing, 211816 P. R. China

## Abstract

The present work investigated the hepatoprotective role of exopolysaccharides (EPS) isolated from the mushroom *Pleurotus geesteranus* with respect to alcohol-induced liver injury in mice. Based on a physico-chemical analysis, the EPS produced by *Pleurotus geesteranus* was identified as a heteropolysaccharide with α-glycosidic bond. The results revealed that prophylactic application of the EPS reduces detrimental alcoholic effects on the liver. This observation was followed by decreased levels of total cholesterol, triglycerides, CYP2E1 and pro-inflammatory mediators (TNF-α, IL-6, IL-1β, COX-2, NO and iNOS) in the liver homogenates, suggesting that the EPS exhibits anti-inflammatory and hepatoprotective effects. Moreover, the increased activity of hepatic enzymes (superoxide dismutase, glutathione peroxidase and catalase) and reduced lipid peroxidation status indicated that the antioxidative effect of the EPS contributes to alleviation of liver injury. Therefore, this study reports that the EPS produced by *Pleurotus geesteranus* could be considered a potential natural drug or functional food supplement for the prevention of liver damage.

## Introduction

Alcoholic liver disease (ALD) has become an important public health issue around the world, with a death rate of 20%^1–3^. In addition, the incidence of ALD has increased in China as a consequence of an increased frequency of alcohol consumption^4^. Previous studies have indicated that excessive and consistent alcohol exposure leads to hepatic steatosis, hepatitis, cirrhosis and even liver cancers^5,6^.

Alcohol is rapidly metabolized in the liver, resulting in the production of the highly toxic acetaldehyde^7^. Alcohol is converted to acetaldehyde mainly by alcohol dehydrogenase (ADH) and is further oxidized to acetate by aldehyde dehydrogenase (ALDH)^8,9^. Cytochrome P450 enzymes, especially the CYP2E1 isoform, represent a secondary pathway responsible for alcohol removal at high concentrations. Breakdown of acetaldehyde by CYP2E1 and ALDH in the mitochondria generates reactive oxygen/nitrogen species, which disturbs the redox homeostasis and causes oxidative stress^10,11^. These free radicals can increase lipid peroxidation by reacting with cellular unsaturated lipids, leading to hepatocyte apoptosis and necrosis, which ultimately results in liver injury^12^.

Reactive oxygen species (ROS) are efficiently eliminated by antioxidative defence systems, which involve enzymes that detoxify oxygen free radicals, such as catalase (CAT), glutathione peroxidase (GSH-Px) and superoxide dismutase (SOD) under normal physiological conditions^13^. However, under pathological conditions, the excess of ROS can lead to apoptosis by activating pro-inflammatory cytokines, for instance tumour necrosis factor-α (TNF-α) and interleukins (IL-1β and IL-6)^14^. Similar to oxidative stress, inflammation is generally involved in the whole spectrum of liver diseases from the initial to the advanced stage^15^. Interestingly, the overexpression of pro-inflammatory cytokines further accelerates the accumulation of ROS, leading to cell damage^16,17^. In addition, induced expression of cyclooxygenase-2 (COX-2) contributes to the inflammatory response and participates in the aggravation of alcohol-induced liver injury^18^. Moreover, nitric oxide (NO) is involved in a wide range of toxic oxidative reactions^1^, and alcohol consumption induces a significant increase in NO levels, as a result of the elevated synthesis of inducible nitric oxide synthase (iNOS)^2^. Therefore, increases in NO generation may be an early indicator of ethanol-induced liver damage, and inhibiting the release of NO is a potential method for controlling inflammation^19^.

The most common medications for ALD are agents that manage and improve alcohol metabolism^20^, such as bifendate, tiopronin and potenline, and are available for clinical treatment against hepatic damage. However, the associated side effects, including dizziness, emesis, diarrhoea, dermatitis and leukopenia, severely limit the applications^1^. Hence, researchers are eagerly exploring natural and non-toxic hepatoprotection agents as potential alternatives for treating alcohol-induced liver injury. In recent years, mushroom-derived natural products have been used as functional foods and represent the basis for the development of new biopharmaceuticals^21^. *Pleurotus geesteranus*, which belongs to phylum *Basidiomycota*, is rich in biologically active components, including polysaccharides, peptides, sterols and dietary fibre^22–24^. As the most abundant metabolites of mushrooms, polysaccharides have gained much attentions owing to various biological activities including hepatoprotective^21^, anti-oxidative^25^, anti-inflammatory^26^, antitumour^27^, antiproliferative^28^ and antihyperlipidaemic effects^29^, *etc*. Based on previous studies by academic researchers, EPS from *P. geesteranus* showed hypolipidaemic, antioxidative and antitumour activities^22,30^. However, the data regarding the chemical structure, hepatoprotective and anti-inflammatory effects of the EPS from *P. geesteranus* are limited.

This work was designed to research the characterization, hepatoprotection and anti-inflammatory effects of an exopolysaccharide from *P. geesteranus* on alcohol-induced liver injury in mice and to identify the underlying hepatoprotective mechanisms for pharmaceutical application.

## Results

### The yield, isolation and purification of the EPS

A crude exopolysaccharide (85.46 g) was obtained from a fermentation broth, and the pure exopolysaccharide weighed 68.15 g after purification. Therefore, the exopolysaccharide yield was 79.74%.

**Figure 1:**
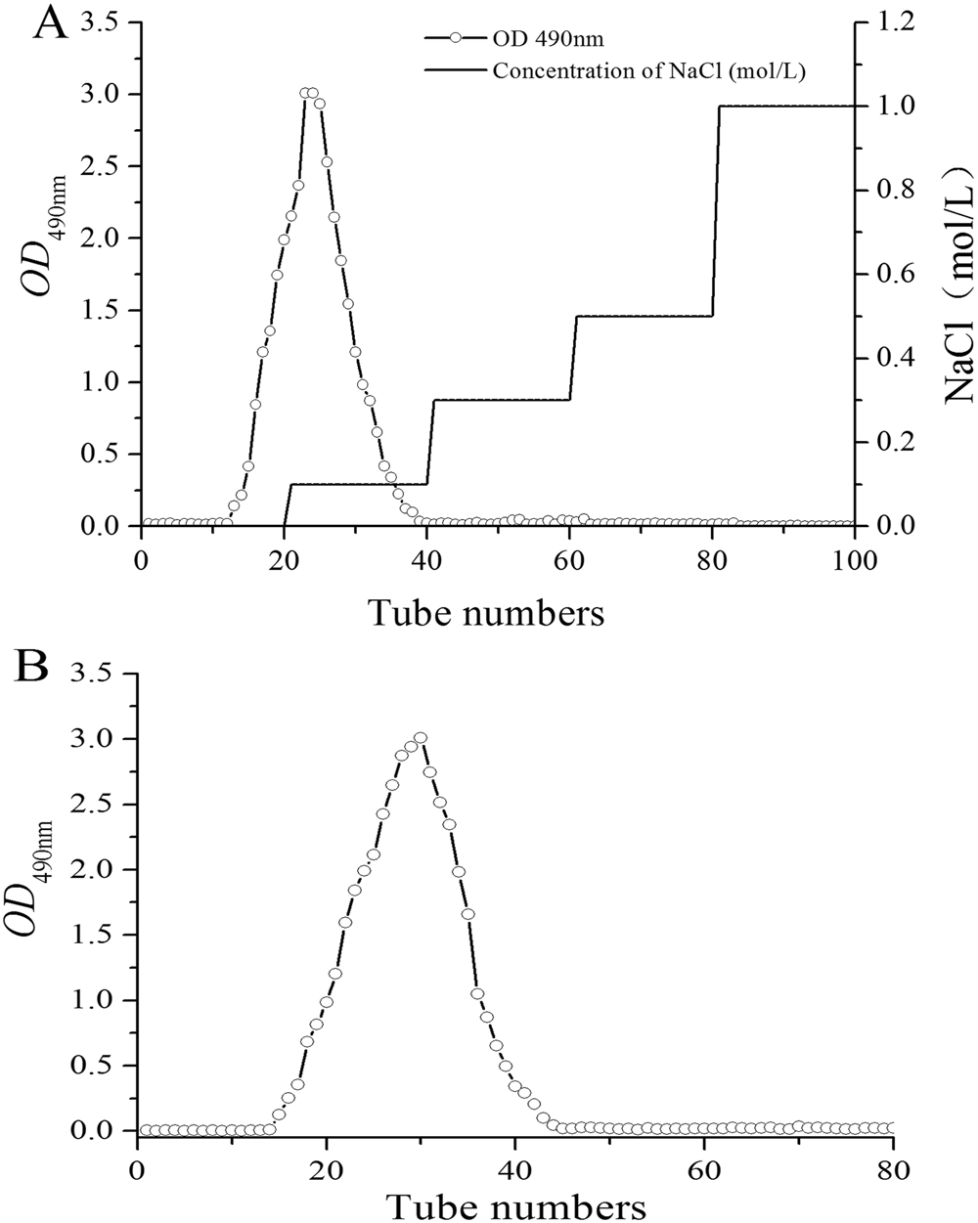
Purification of EPS. (**A**) Elution profile on DEAE-52 cellulose column chromatography with gradient of NaCl solution. (**B**) Elution profile on Sephadex G-100 cellulose column chromatography with distilled water.

The elution profile of EPS by DEAE-52 and Sephadex G-100 cellulose column chromatography is illustrated in Fig. 1. Obviously, there is only one peak (Fig. 1A), which was purified by DEAE-52 cellulose column chromatography, and the EPS consisted of neutral polysaccharides since it was eluted by distilled water^31^. The fractions were further purified by a Sephadex G-100 cellulose column, and the purified subfractions revealed a single and relatively symmetrical peak (Fig. 1B).

### Structural characterizations

**Figure 2:**
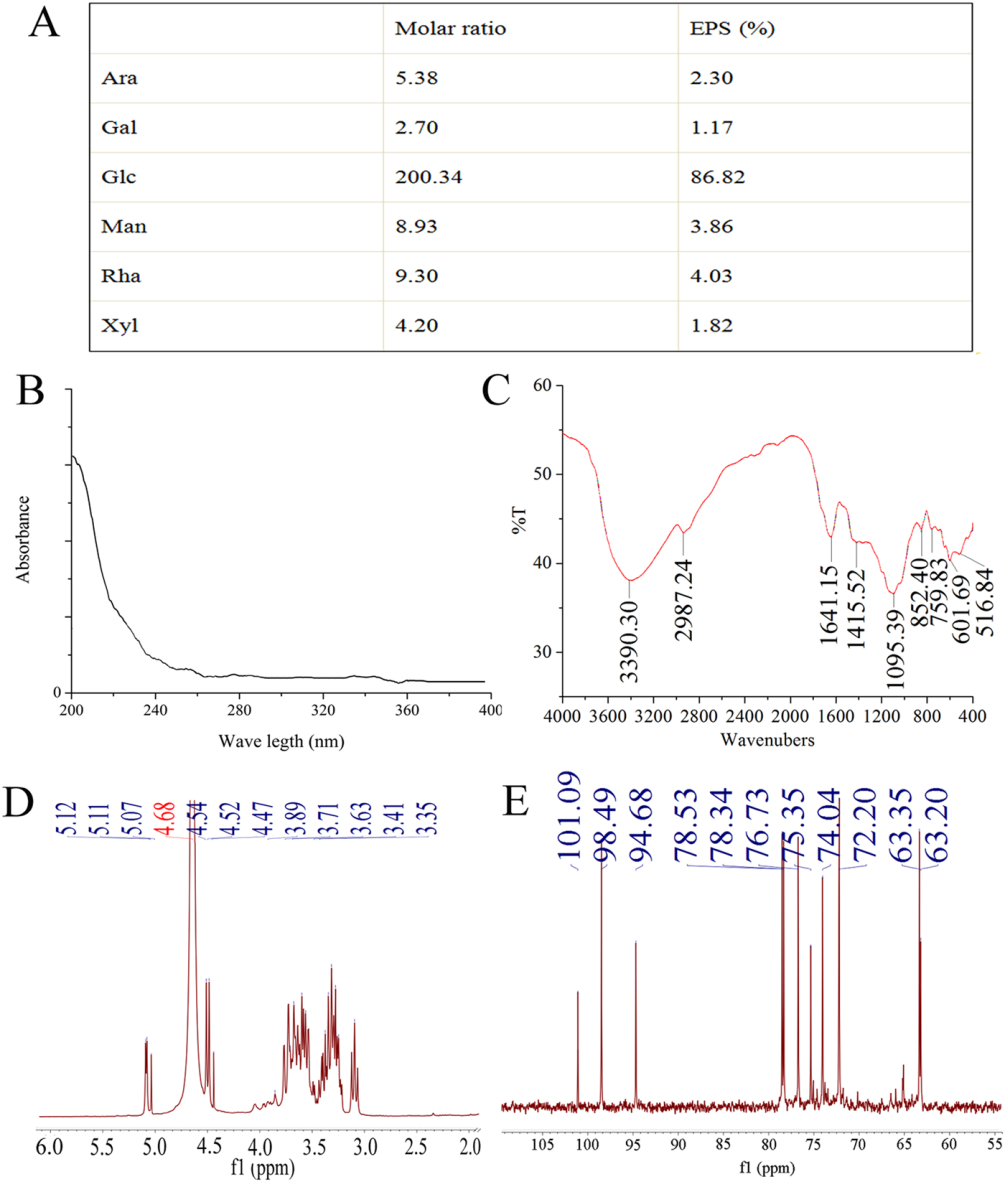
Physicochemical analysis of EPS. (**A**) Gas chromatographs; (**B**) UV analysis; (**C**) FTIR spectra analysis; (**D**,**E**) NMR analysis of EPS for ^1^H spectra and ^13^C spectra.

Compared the retention times with those of standard sugars, the monosaccharide compositions of the EPS were identified by gas chromatography (GC) analysis (Fig. 2A). The EPS included six of the following monosaccharides: arabinose (Ara), galactose (Gal), glucose (Glc), mannose (Man), rhamnose (Rha) and xylose (Xyl).

The purity of EPS was analysed by an ultraviolet (UV) spectrophotometer, and the result is shown in Fig. 2B. The lack of obvious absorption at 260 and 280 nm indicated that there were no proteins or nucleic acids in the exopolysaccharides.

The Fourier transform infrared (FTIR) spectroscopy of the EPS is shown in Fig. 2C. In detail, the characteristic bands at approximately 3,400 cm^−1^ were with the O-H stretching vibrations^32^, and the presence of an absorption band at approximately 2,987 and 1,415 cm^−1^ indicated the existence of carbohydrate^33,34^. Furthermore, the sharp bands at 1,641 cm^−1^ could be correlated with the stretching vibration of the carbonyl group^29^. Additionally, the strong absorption peaks of 1,095 cm^−1^ indicated the possible presence of a furanose ring in the polysaccharides^35^, and the weak absorption peak at approximately 850 cm^−1^ indicated the existence of α-glycosidic bond in the EPS^33^.

Further evidence for the formation of the EPS was provided by the ^13^C nuclear magnetic resonance (NMR) and ^1^H NMR spectra analysis, and the signal at approximately δ 4.68 (^1^H NMR) was assigned to the internal acetone in D2O solvent. As shown in Fig. 2D, the ^1^H NMR spectrum contained six anomeric protons signals at δ 5.12, δ 5.11, δ 5.07, δ 4.54, δ 4.52 and δ 4.47, showing that the EPS was mainly composed of six types of monosaccharides^36^, and the results were in accordance with the GC analysis. Moreover, the ^1^H NMR spectrum of the EPS exhibited a set of consecutive signals (δ 3.0–4.0) due to the CH_2_-O and CH-O groups of sugars^37^. The anomeric proton resonances were over 5.0 ppm, making clear that the glucosyl linkage was in the α-form^38^, which was consistent with the analysis of the FTIR spectrum. Moreover, in the anomeric region of the ^13^C NMR spectrum of EPS (Fig. 2E), the anomeric carbon (C1) signals of glycosides were assigned to 100–104 ppm, and C2, C3, C4, C5 and C6 from the glycosidic ring were assigned to 60–80 ppm, which were the general representative carbohydrate peaks^37,38^. Furthermore, the ^13^C NMR chemical shift of the EPS was between 98 ppm and 102 ppm^33^, indicating that the glucosyl linkage was in the α- configurations, which was consistent with the analysis of the ^1^H NMR and FTIR spectrum.

### Ultrastructures of the EPS

**Figure 3:**
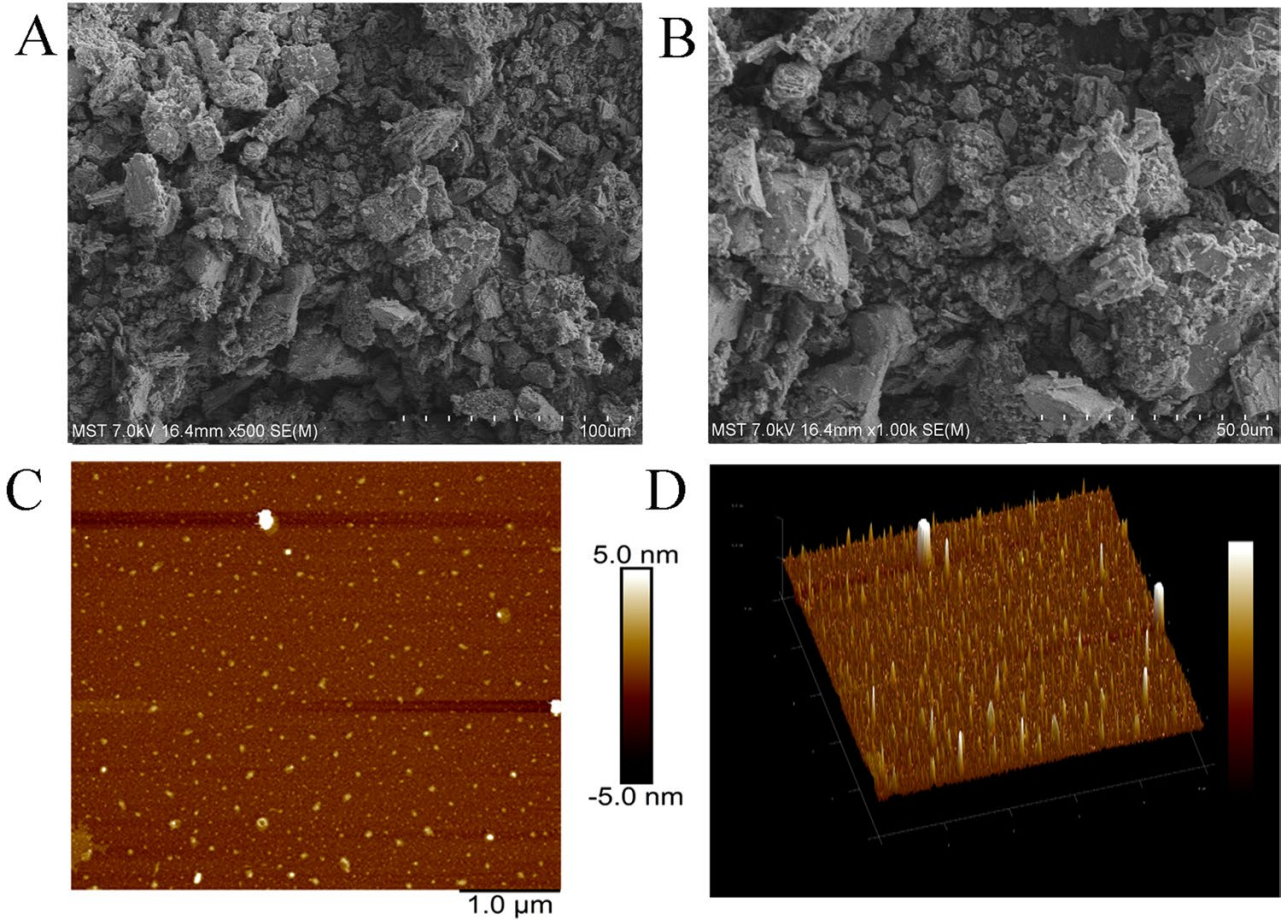
Ultrastructures analysis of EPS. (**A**) SEM images of EPS (500x); (**B**) SEM images of EPS (1,000x); (**C**) AFM aeroview images of EPS; (**D**) AFM stereogram images of EPS.

To analyse the conformation of individual macromolecules, a scanning electron microscope (SEM) is mostly used for the imaging of exopolysaccharides^27^. As shown in Fig. 3A,B, the EPS had a rough surface with an island-like appearance, which consisted of many small lumpish particles and irregular pores, and differed from the exopolysaccharide of *Schizophyllum commune* reported by Du *et al*.^26^, in which the surface of the exopolysaccharide was smooth and had a glittering surface. Atomic force microscopy (AFM) has also been a valuable metrological tool to study the spatial structure and surface morphology of biomacromolecules on the nanometre scale of particles^33^. The AFM images of EPS with different conformations are illustrated in Fig. 3C,D. The EPS aggregated to form tree-like lumps with a diameter ranging from 100 to 400 nm and a height ranging from 1.0 to 5.0 nm, which was different from the polysaccharide from *Oudemansiella radicata* reported by Gao *et al*.^33^.

### Effects of EPS on the body weights and liver indexes

The effects of EPS on the body weights and liver indexes in alcohol-induced liver injury mice are presented in Table 1. Obviously, there were no significant differences in initial body weights in the mice, but the weight gained in the NC group was significantly higher than that in the MC group (*P* < 0.05) at the end of the experiment. With administration of the EPS in mice at the high dose (600 mg/kg), the body weight was increased by 56.23%, while it was 49.40% higher at the low dose (200 mg/kg) when compared with the body weight in the MC group (35.87%). After the treatment of EPS, the liver indices were decreased by 34.55, 30.96 and 24.35% at doses of 600, 400 and 200 mg/kg when compared with those in the MC group, respectively, indicating that the EPS was capable of significantly repressing the liver index gain induced by alcohol. Additionally, the body weight was significant increased by 43.52% and the liver index was decreased by 21.32% in the PC group, in comparison with the values in the MC group.

**Table 1:** Effects of EPS on body weights and liver indexes on ALD.

Groups	Body weights (g)		Liver weights (g)	Liver index (%)
	Initial	Final		
NC	24.32 ± 2.77	36.68 ± 2.17	3.15 ± 0.13	8.59 ± 0.38
MC	25.90 ± 2.01	35.19 ± 2.11 d	5.00 ± 0.11 d	14.21 ± 1.03 d
PC	25.62 ± 2.62	36.77 ± 2.09 c	4.11 ± 0.13 c	11.18 ± 0.77 c
**EPS**				
600 mg/kg	25.59 ± 2.23	39.98 ± 2.27 b	3.72 ± 0.15 b	9.30 ± 0.24 b
400 mg/kg	25.72 ± 2.42	39.15 ± 2.32 b	3.84 ± 0.14 b	9.81 ± 0.33 b
200 mg/kg	25.65 ± 2.11	38.32 ± 2.15 b	4.12 ± 0.13 c	10.75 ± 0.42 c

The values were reported as the Mean ± S.D. of ten mice in each group. Means with the same letter are not significantly different (*P* < 0.05, one-way ANOVA followed by Duncan’s post-hoc test).

(d)*P* < 0.01, statistically significant compared with NC group.

(c)*P* < 0.01, statistically significant compared with MC group.

(b)*P* < 0.05, statistically significant compared with MC group.

### Effects of EPS on hepatic lipid levels and enzyme activities

**Figure 4:**
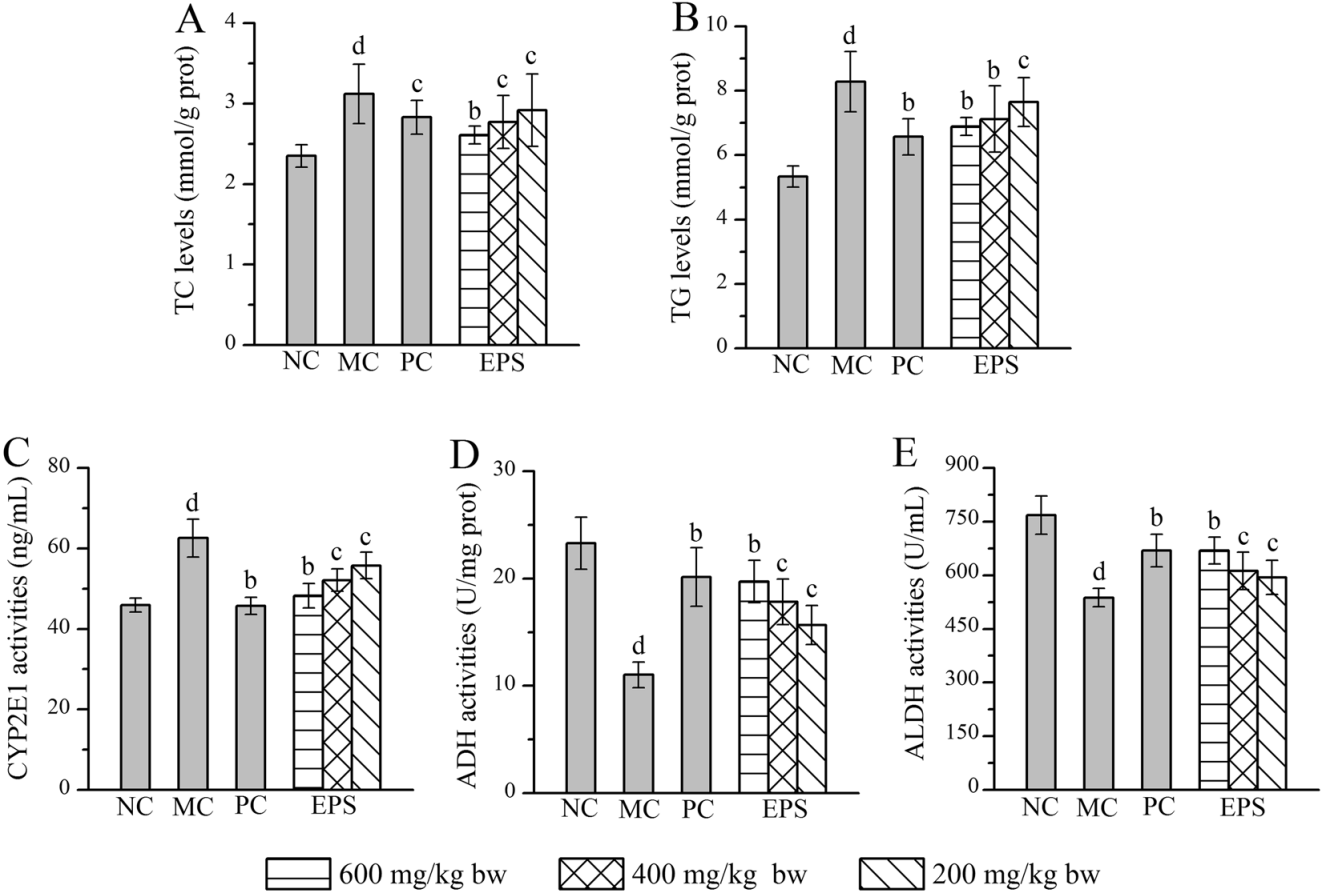
Effects of EPS on hepatic lipid levels and enzyme activities. (**A**) TC; (**B**) TG; (**C**) CYP2E1; (**D**) ADH; and (**E**) ALDH. The values were reported as the Mean ± S.D. (n = 10 for each group). Means with the same letter are not significantly different (*P* < 0.05, one-way ANOVA followed by Duncan’s post-hoc test). (d) *P* < 0.01, statistically significant compared with NC group; (c) *P* < 0.01, statistically significant compared with MC group; (b) *P* < 0.05, statistically significant compared with MC group.

The hepatic lipid levels (total cholesterol (TC) and triglyceride (TG)) and hepatic enzyme activities (CYP2E1, ADH and ALDH) in alcohol-induced liver injury mice treated with EPS are presented in Fig. 4. Mice in the MC group showed significant increases in TC, TG and CYP2E1 and decreases in ADH and ALDH (with all *P* < 0.05) when compared with the NC group, which provided the evidence of hepatocyte damage induced by alcohol. Briefly, the hepatic TC levels decreased by 16.35, 11.22 and 6.41% with the EPS treatment at the three doses (600, 400 and 200 mg/kg), while the hepatic TG levels were 16.79, 14.01 and 7.61% lower than those in the MC group, respectively. Moreover, the CYP2E1 activities decreased to 48.26 ± 1.54 ng/mL by the treatment with EPS at the dose of 600 mg/kg, which was significant lower than that in the MC group, and the ADH and ALDH activities were increased to 19.73 ± 0.88 U/mg protein and 669.35 ± 18.71 U/mL, respectively, by treatment with EPS at the high dose (600 mg/kg), and these activities were significantly higher than those in the MC group. Similar conclusions can be drawn when compared with the results in other dose groups. Furthermore, the PC group (bifendate-treated mice) at a dose of 150 mg/kg also showed obvious decreases in TC, TG and CYP2E1, as well as increases in ADH and ALDH activities compared with the levels in the MC group.

### Effects of EPS on inflammatory mediators

**Figure 5:**
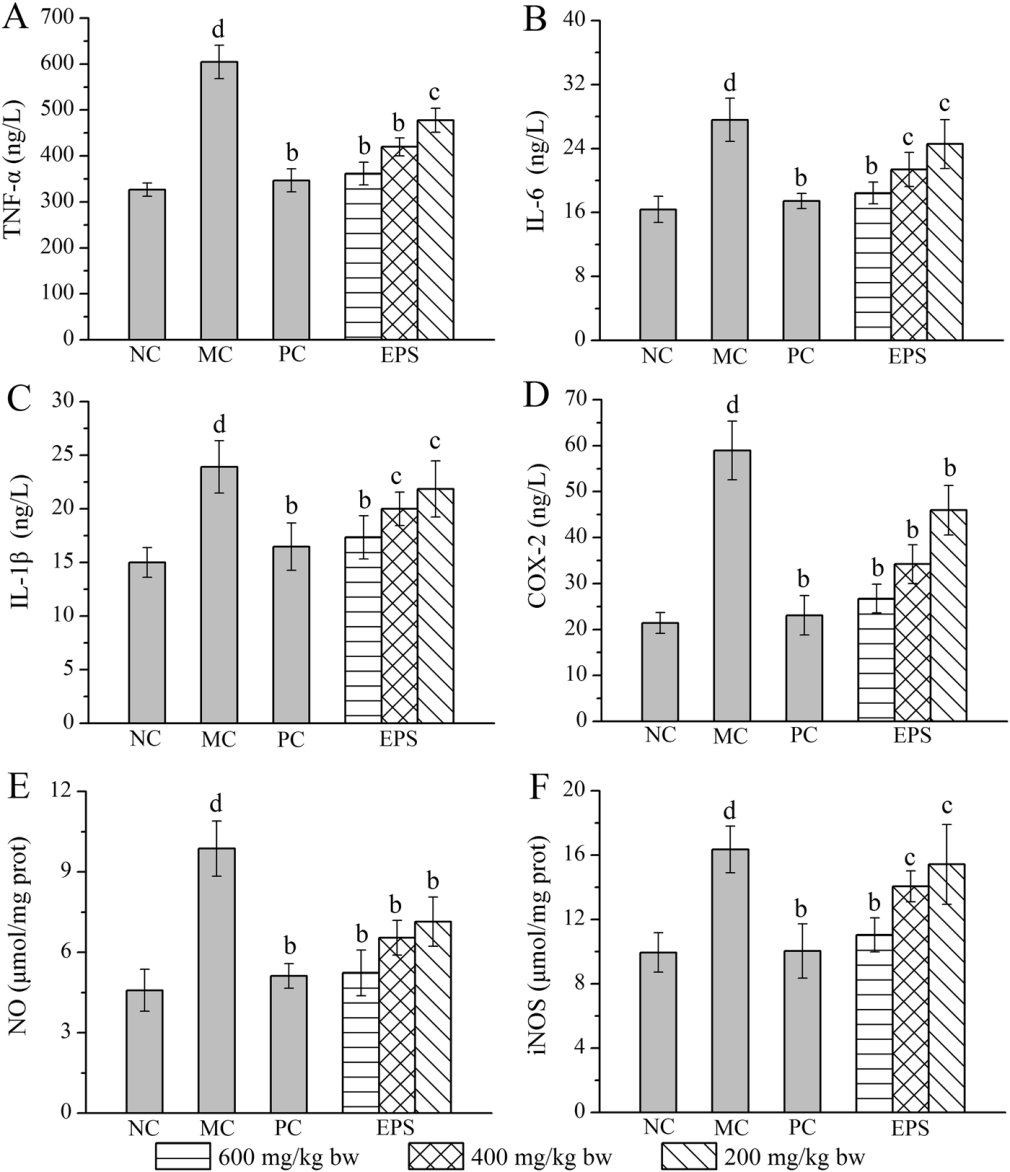
Effects of EPS on levels of pro-inflammatory mediators. (**A**) TNF-α; (**B**) IL-6; (**C**) IL-1β; (**D**) COX-2; (**E**) NO; and (**F**) iNOS. The values were reported as the Mean ± S.D. (n = 10 for each group). Means with the same letter are not significantly different (*P* < 0.05, one-way ANOVA followed by Duncan’s post-hoc test). (d) *P* < 0.01, statistically significant compared with NC group; (c) *P* < 0.01, statistically significant compared with MC group; (b) *P* < 0.05, statistically significant compared with MC group.

The levels of six important inflammatory mediators (TNF-α, IL-6, IL-1β, COX-2, NO and iNOS) in the liver homogenate were investigated to prove EPS has anti-inflammatory effects. As shown in Fig. 5, the levels of TNF-α, IL-6, IL-1β, COX-2, NO and iNOS in the MC group were significantly increased when compared with those in the NC group (with all *P* < 0.05), indicating that the inflammatory reaction had appeared in the liver. However, the TNF-α, IL-6 and IL-1β levels reached a minimum with decreasing rates of 40.20, 33.16 and 27.48% after the pre-treatment with EPS at the dose of 600 mg/kg (Fig. 5A–C), which compared with those in the MC group, but were decreased by 21.02, 10.95 and 8.62% at the dose of 200 mg/kg. Moreover, the COX-2, NO and iNOS levels decreased by 54.70, 47.01 and 32.52%, respectively, with the EPS treatment at the dose of 600 mg/kg, which were lower than the levels in the MC group. Similar conclusions could be drawn by the same methods in the other doses and PC groups.

### Effects of EPS on antioxidative status and serum biochemistry

**Figure 6:**
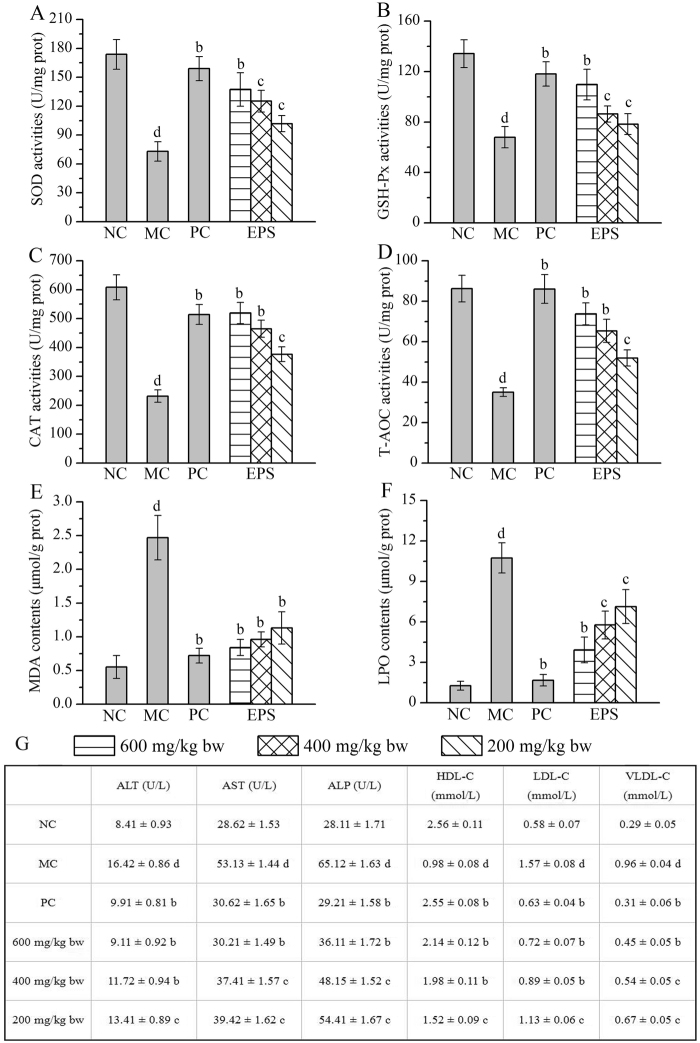
Effects of EPS on antioxidative status and serum biochemistry. (**A**) SOD; (**B**) GSH-Px; (**C**) CAT; (**D**) T-AOC; (**E**) MDA; (**F**) LPO of hepatic antioxidative enzymes; and (**G**) Effects of EPS on serum activities. The values were reported as the Mean ± S.D. (n = 10 for each group). Means with the same letter are not significantly different (*P* < 0.05, one-way ANOVA followed by Duncan’s post-hoc test). (d) *P* < 0.01, statistically significant compared with NC group; (c) *P* < 0.01, statistically significant compared with MC group; (b) *P* < 0.05, statistically significant compared with MC group.

Several antioxidative enzymes and substances in the liver homogenate were provided evidences as biochemical markers for early liver damage. As shown in Fig. 6A–E, decreases in hepatic antioxidative enzyme activities (SOD, GSH-Px, CAT and total antioxidant capacity (T-AOC)), as well as increases in the lipid product contents (malondialdehyde (MDA) and lipid peroxidation (LPO)) were observed in MC mice compared with those in the NC group, revealing that the injection of alcohol induced serious oxidative stress. The SOD, GSH-Px, CAT and T-AOC activities were significantly increased by 88.21, 61.54, 124.09 and 110.50% at the high dose (600 mg/kg), by 71.52, 27.12, 100.58 and 86.42% at the middle dose (400 mg/kg), and by 39.54, 15.27, 62.44 and 48.25% at the low dose (200 mg/kg), respectively. These activities were significantly higher than the respective activities in the MC group (with all *P* < 0.05). In addition, the hepatic MDA and LPO contents were decreased by 65.99 and 63.50% in the EPS-treated mice (600 mg/kg), compared with the contents in the MC group. Similar tendencies of the MDA and LPO activities at the 400 and 200 mg/kg EPS doses could also be observed in the PC group.

Additionally, several serum enzymes and serum lipoproteins were used as biochemical markers for liver damage. As shown in Fig. 6G, the serum activities of alanine aminotransferase (ALT), aspartate aminotransferase (AST) and alkaline phosphatase (ALP), as well as the levels of low-density lipoprotein cholesterol (LDL-C) and very-low-density lipoprotein cholesterol (VLDL-C) were significantly increased, while the high-density lipoprotein cholesterol (HDL-C) levels were significantly decreased in the MC group, compared with the levels in the NC group, which was the proof of hepatocyte damage. The ALT, AST and ALP activities reached 9.11 ± 0.92, 30.21 ± 1.49 and 36.11 ± 1.72 U/L at the dose of 600 mg/kg, respectively, which were significantly lower than the activities in the MC group (with all *P* < 0.05). Furthermore, after the gavage administration with EPS at the dose of 600 mg/kg, the HDL-C level reached 2.14 ± 0.12 mmol/L, which was higher than that in the MC group, while the LDL-C and the VLDL-C levels reached 0.72 ± 0.07 and 0.45 ± 0.04 mmol/L, respectively, which were all significantly lower than the levels in the MC group (with all *P* < 0.05). Additionally, compared with the MC group, the bifendate-treated mice (PC group) also showed significant therapeutic effects inhibiting the enhancement of MDA, LPO, ALT, AST, ALP, LDL-C and VLDL-C and the reduction of SOD, GSH-Px, CAT, T-AOC and HDL-C in the serum.

### Histopathological observations

**Figure 7:**
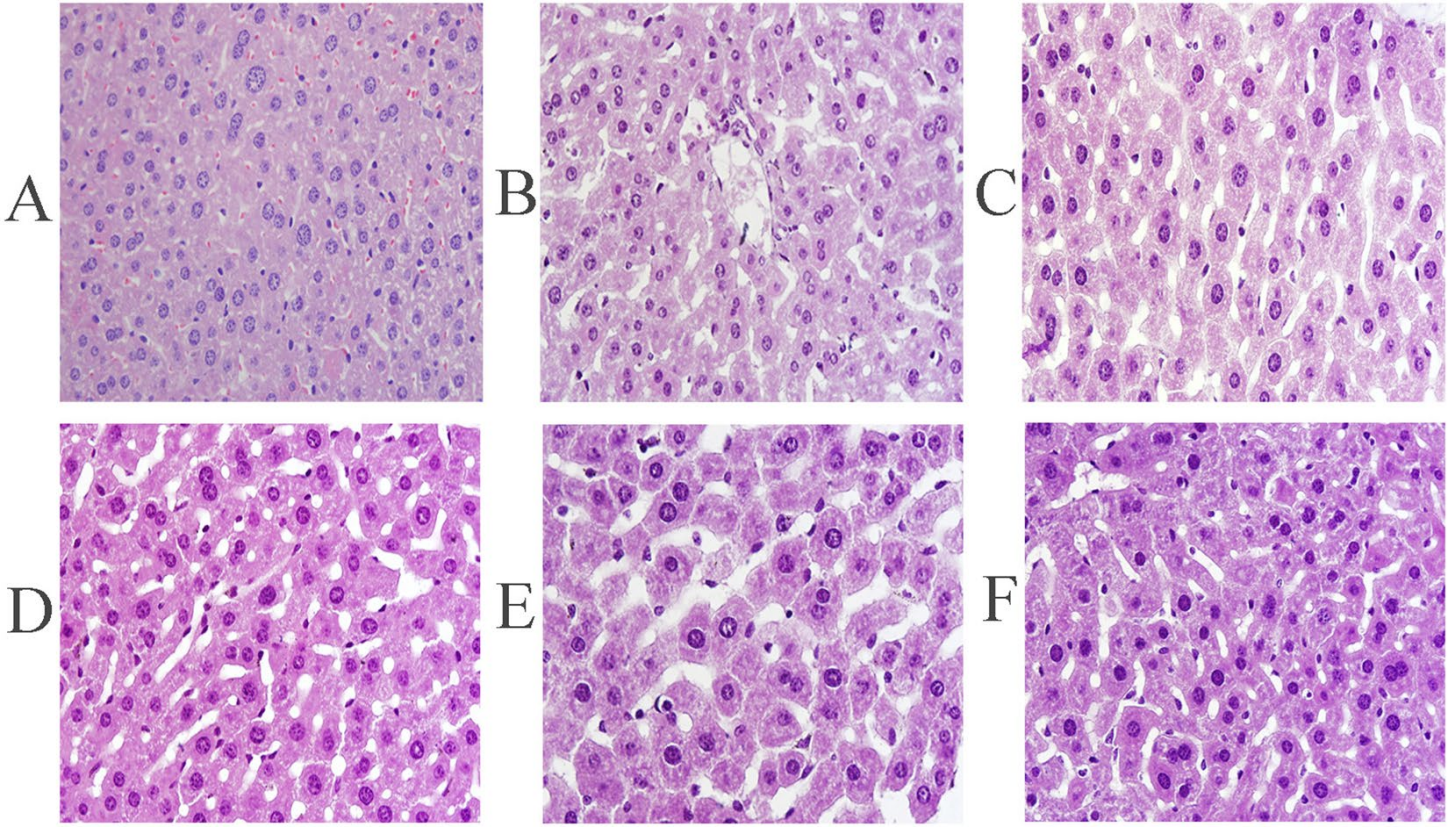
Representative photomicrographs of liver histopathology (600×). Liver sections from mice in (**A**) NC group, (**B**) MC group, (**C**) PC group, and dosage groups treated with EPS at the dose of (**D**) 600 mg/kg and (**E**) 400 mg/kg and (**F**)200 mg/kg, respectively.

In the present work, histopathological observations of the liver were performed to corroborate the evidence from the biochemical analysis (Fig. 7). We observed a regular hepatocyte morphology, orderly arranged hepatic cell, unbroken cell borders, and no inflammation and cell infiltration (score = 0) in the NC group (Fig. 7A). In contrast, alcohol-injection (MC group) caused severe liver damage as characterized by cell ballooning degeneration, loss of cellular boundaries, hepatic cell necrosis and other inflammatory changes (score = 4) (Fig. 7B). After the treatment with EPS and bifendate, the mice exhibited improved histopathological results, which were evidenced by the decreases in necrotic zones, integrity of cellular boundaries, and decreases in inflammatory cells. According to our scoring system, the scores of the treatment groups were 1 (PC group), 2 (600 mg/kg), 3 (400 mg/kg), and 3 (200 mg/kg), indicating that the high-dose (600 mg/kg) EPS treatment was better than the others.

### Acute toxicity analysis

In the present study, the mice did not exhibit any gross behavioural changes including respiratory distress, abnormal locomotion, and catalepsy or any toxic symptoms and being shaggy-haired after treatment with EPS, either immediately or during the post-treatment period (14 days), even at the dose of 4,000 mg/kg compared to the control, indicating that the EPS was practically a non-toxic substance^39^.

## Discussion

Prior work has documented the effectiveness of polysaccharide intervention in improving liver injury and reducing oxidative stress. Liu *et al*.^1^ reported that the polysaccharides from *Antrodia cinnamomea* had significant hepatoprotective effects on acute alcohol-induced liver injury in mice. In addition, Gao *et al*. reported the anti-inflammatory and renoprotective effects of selenized mycelial polysaccharides from *Oudemansiella radicata*^33^, and Du *et al*.^26^ and Dinić *et al*.^40^ reported the anti-inflammatory potential of exopolysaccharide. However, few studies have focused on exopolysaccharides for treating alcoholic liver diseases or have described the physicochemical characterization. In this work, we evaluated the antioxidative, anti-inflammatory and hepatoprotective effects of an EPS from *P. geesteranus*. We found that EPS supplementation yielded potential hepatoprotective effects reflected by a decrease in TC, TG, CYP2E1 and inflammatory mediators (TNF-α, IL-6, IL-1β, COX-2, NO and iNOS), increasing ADH, ALDH and the hepatic enzyme activities of SOD, GSH-Px, CAT and T-AOC as well as down-regulating the contents of MDA and LPO. Moreover, the hepatoprotective and anti-inflammatory effects of EPS against alcohol-induced liver injury have been directly certified by improved necrotic accumulation, inflammatory infiltration and fatty droplet formation in the liver. These findings were consistent with those of Wang *et al*.^4^ and Liu *et al*.^12^ have reported, indicating that the EPS have potential antioxidative, anti-inflammatory and hepatoprotective effects against ALD.

Additionally, exopolysaccharides were obtained by submerged fermentation and showed advantages superior to other polysaccharides extracted from the fruiting body, mycelial polysaccharides and others^40,41^, including a low cost, a short processing time, ease of purification, and higher yields^42^. The possible reasons may be related to the composition of the different strains and culture media and the different conditions of fermentation. In addition, this may be the reason why the present physicochemical characterizations were different from those in the other studies. In our work, we analyzed the physicochemical characterizations by GC, FTIR, NMR, and the structural analysis revealed the existence of an α-glycosidic bond and abundant glucose in the EPS, the type of bond was different from Du *et al*. reported^26^, and the sugar content was different from Gao *et al*. reported^33^. Additionally, we observed the rough surface of EPS *via* the ultrastructures revealed by the SEM and AFM, and more differences were reported by Gao *et al*.^33^.

Previous studies have demonstrated that oxidative stress is a primary pathological mechanism of alcohol-induced liver injury, and in particular, ROS-induced oxidative stress can generate toxic lipid intermediates and lead to liver damages under pathological conditions^4,25^. Moreover, the superfluous lipid intermediates (MDA and LPO) may disturb the hepatic antioxidative defence^20^; consequently, hepatic MDA and LPO formation have been commonly used as indicators of liver damage. Antioxidative enzymes such as SOD can promote the conversion of superoxide radicals to hydrogen peroxide^25^, and the hydrogen peroxide (H_2_O_2_) is decomposed into water and oxygen by GSH-Px and CAT, resulting in the prevention of ROS formation^33^. EPS not only suppressed the overproduction of ROS but also enhanced the activities of SOD, CAT, GSH-Px and T-AOC and decreased the levels of MDA and LPO. The restoration of SOD, CAT, GSH-Px and T-AOC activity ameliorates alcohol-induced liver injury by reducing oxidative stress. Our data from the mouse model of alcohol-induced liver injury suggest that the antioxidative activities of EPS are crucial to its hepatoprotective effect.

Inflammation is another representative pathological mechanism that is responsible for alcohol-induced liver injury^10^. Oxidative stress can induce the expression of pro-inflammatory cytokines to facilitate inflammatory responses, which aggravates liver injury^43^. TNF-𝛼 is a representative inflammatory cytokine that is closely associated with the progression of many inflammatory diseases^26,44^. Moreover, the augmented production of iNOS is also attributed to the development of hepatic injury because such production can induce the formation of NO, which is another factor that is involved in oxidative stress and the innate immune system, which in turn are involved in inflammation^1,19^. The upregulation of inducible COX-2 expression is an important aspect of inflammatory responses and participates in the augmentation of alcohol-induced liver injury^45^. Alcohol stimulates the production of TNF-𝛼, IL-1β, IL-6 and COX-2, which can increase the release of proteolytic enzymes from neutrophils, and lead to accumulation of ROS^18^. In turn, oxidative stress leads to inflammation^1^. In fact, in our investigation, the levels of TNF-α, IL-1β, IL-6, COX-2, NO and iNOS in the liver homogenates were decreased with the EPS treatment, attesting that the EPS from *P. geesteranus* plays an important role in hepatoprotection.

In addition to the above two mechanisms, CYP2E1, ADH and ALDH were also pivotal factors in the regulation of alcohol-induced liver injury as a compensatory mechanism^46^. The metabolism of alcohol contains two steps: first, alcohol is converted to acetaldehyde primarily by ADH, and second, acetaldehyde is further metabolized to acetate by ALDH. Acetaldehyde is a toxic intermediate that interacts with lipids to cause free radical formation and cell damage^7,8^. During the activation of ADH and ALDH by alcohol consumption, CYP2E1 (the major hepatic metabolic enzyme in liver microsomes) can release ROS while participating in alcohol metabolism, which causes damage to the liver^11,47^. These lines of evidence suggest that CYP2E1 plays a critical role in increased oxidative stress; therefore, it is possible that the hepatoprotective effect against alcohol might be attributed to counteracting oxidative stress *via* inhibition of CYP2E1^10,46^. The present results revealed that EPS treatment inhibited CYP2E1 activities and enhanced ADH and ALDH activities compared with the activities in the MC group, indicating that EPS might exert its hepatoprotective effects by stimulating ethanol metabolism.

In addition, several studies have reported that high-levels of TC and TG in the liver can increase the liver lipid contents, potentially inducing the development of liver diseases^21^. *In vivo*, HDL (a “beneficial” lipoprotein for health) particles carry the TC to the liver from peripheral tissues and cells for catabolism through the blood circulation, thereby reducing the risk of TC *via* the “reverse cholesterol transport” pathway^39,48^. However, LDL-C and VLDL-C as the main carriers can inhibit the transport of TC and thus induce the atherosclerotic lesions in the blood vessel walls, which leads to increased levels of oxidative stress^29^. Moreover, high TC and TG levels may increase the blood viscosity, potentially inducing the development of liver injury^21,49^. Therefore, low-level HDL-C and high-level TC, TG and LDL-C are harmful for health. In the present work, the EPS treatments showed effects in lowering the TC, TG, LDL-C and VLDL-C levels and elevating the HDL-C levels in ALD mice.

## Materials and Methods

### Chemicals and reagents

The DEAE-52 cellulose and Sephadex G-200 were purchased from Sigma Chemicals Company (St. Louis, USA). The diagnostic kits using a spectrophotometric method for investigating the activities of CYP2E1, ADH, ALDH, SOD, CAT, GSH-Px and T-AOC as well as contents of TC, TG, LPO and MDA were purchased from Nanjing Jiancheng Bioengineering Institute (Nanjing, China). The enzyme-linked immunosorbent assay (ELISA) diagnostic kits for TNF-α, IL-1β, IL-6, COX-2, NO and iNOS investigation were supplied by Jiangsu Meibiao Biological Technology Company, Limited (Jiangsu, China). The standard monosaccharaides, including Rha, ribose (Rib), Ara, Xyl, Glc, fucose (Fuc), Man and Gal, were purchased from Sigma Chemicals Company (St. Louis, USA). All other reagents and chemicals used in the present work were analytical grade and were provided by local chemical suppliers in China.

### Preparation of EPS

The strain of *P. geesteranus* used in the present work was preserved in the Fungi and Application Laboratory of Shandong Agriculture University (Tai’an, China). The preparation of EPS was referenced to Song et al. reported with slight modification^39^. The seedling cultivation was performed in a 1 L filter flask containing 500 mL of potato (200 g/L), glucose (20 g/L), KH_2_PO_4_ (1.5 g/L) and MgSO_4_ × 7H_2_O (1 g/L). The batch fermentation conditions, including the temperature (25 °C), pH (7.0) and air supply, were controlled automatically by a fermenter (100-L, Xianmin, China). The *P. geesteranus* fermentation broth was isolated by filtration and concentrated with an Electro-Thermostatic Blast Oven (DHG-9143B5-III, Shanghai, China) at 60 °C. After centrifugation (3,000 × g, 15 min), the supernatant fermentation broth was mixed with three volumes of ethanol (95%, v/v) and incubated at 4 °C overnight^21,29^. The precipitate was deproteinized with Sevag reagent (chloroform/n-butanol, 5:1, v/v)^50^ and lyophilized by vacuum freeze-drying (Labconco, USA) to obtain the EPS. The EPS was weighed.

### Purification of EPS

The EPS was purified by DEAE-52 cellulose column chromatography according to the method of Du *et al*.^26^, with a slight modification. EPS (2.0 g) was dissolved in 30 mL of deionized water (70 °C) and filtered through an organic membrane filter. Then, the EPS was fractionated by a DEAE-52 cellulose column (26 mm × 400 mm); the column was stepwise eluted with distilled water and different concentrations of NaCl solutions (0, 0.2, 0.3, 0.5 and 1.0 mol/L NaCl) with a flow rate of 2.0 mL/min. The eluate was collected automatically (2 mL/tube) through a fraction collector (BSZ-100, Huxi, Shanghai, China), and the carbohydrate content in each tube was analysed spectrophotometrically using a phenol-sulfuric acid method^51^.

After dialyzing and concentrating, the eluted fractions were further loaded onto a Sephadex G-100 column (26 mm × 600 mm) and eluted with deionized water at a flow rate of 1.0 mL/min. After dialysis with distilled water for 12 h, the major eluates were collected, concentrated and lyophilized for further experiments.

### Structural characterization of EPS

#### UV analysis

UV spectra were implemented as previously described^33^. The ultraviolet spectrum of the polysaccharide samples with a final concentration of 0.1% was recorded with an ultraviolet spectrophotometer (Shanghai, China) in the range of 200–400 nm.

#### Monosaccharide composition analysis

The method of GC was referenced to Song et al. reported with slight modification^39^.The monosaccharide compositions were analysed by GC (GC-2010, Shimadzu, Japan) equipped with a capillary column of Rtx-1 (30 m × 0.32 mm × 0.2 μm) using the published method^32^. The supernate (1 μL) was injected into the column for analysation after hydrolysation and acetylation. The monosaccharide identifications were processed by comparison with the standard sugars of Rha, Fuc, Rib, Ara, Xyl, Man, Gal and Glc.

#### FTIR spectra analysis

The polysaccharide samples (1 mg) were mixed with KBr powder (100–200 mg) and then pressed into pellets for infrared spectral analysis within a range of 4,000–400 cm^−1^. The FTIR spectrum of the polysaccharide was measured by a spectrophotometer (Nicolet 8700, Nicolet, USA)^34,39^.

#### NMR analysis

^1^H and ^13^C NMR measurements were conducted using a Bruker AV-300 spectrometer operating at 300 MHz at 25 °C, and the sample was dissolved in deuterated water (D_2_O)^39,52^.

#### SEM analysis

The morphologies of EPS were obtained by using cold field emission scanning electron microscopy (S4800, Japan). The powdered sample was directly mounted on a metal stub and then sputtered with gold powder. Finally, the samples were observed with 500- and 1,000-fold magnification at 7.0 kV under a high-vacuum condition. All the measurements were made in ambient conditions^36^.

#### AFM analysis

The ultrastructure of EPS was observed by AFM (BioScope Catalyst NanoScope V, Bruker, Billerica, MA). The samples were dissolved in distilled water (10 μg/mL) and filtered by 0.45 μm filter (NYL, 13 mm syringe filter, Whatman, Inc., USA), and then 5 μL of the diluted solution was dropped onto a freshly cleaved mica substrate and allowed to dry at room temperature. All images were obtained with 256 × 256 pixels at a scanning rate of 1.0 Hz per line, in tapping modes. All the measurements were made in ambient conditions^53^.

### Animal experiments

Kunming strain mice (male, 20 ± 2 g, 8-week-old) purchased from Taibang Biological Products Limited Company (Tai’an, China), were housed in cages under controlled conditions of 12 h light/dark cycles at 22 ± 2 °C and 50–55% humidity, with free access to water and standard food. All experiments were performed in accordance with the Regulations of Experimental Animal Administration issued by the State Committee of Science and Technology of the People’s Republic of China.

The animal experiment was processed using the method reported by Song et al. reported with slight modification^39^. After a week of acclimatization, all mice were randomly divided into six groups (ten mice in each group) including one normal control (NC) group, one model control (MC) group, one positive control (PC) group, and three dose groups treated with EPS (EPS was dissolved in distilled water). During the gavage procedure, the mice in the three dose groups received EPS in doses of 600, 400 and 200 mg/kg, with the use the dose of 150 mg/kg of bifendate in the PC groups and saline water in the NC and MC groups as controls^4,29,39^. The gavage was processed with a syringe by by intragastric administration (i.g.) once daily and was continued for twenty-five successive days. On the twenty-sixth day, all mice except those in the NC group were injected intraperitoneally with alcohol (50%, v/v, 12 mL/kg bw) for induction of ALD^48^. Twelve hours after the alcohol injection, all mice were fasted overnight, weighed and sacrificed by exsanguination *via* anaesthesia.

Blood sample from each mouse was obtained from the retrobulbar vein and centrifuged at 14,000 rpm (4 °C, 10 min) to obtain the required serum. The AST, ALT and ALP, HDL-C, LDL-C and VLDL-C levels were measured using an automatic biochemical analyser (ACE, USA).

The livers were rapidly excised, weighed (the liver index was calculated as liver weight/final body weight × 100) and homogenized (1:9, w/v) in phosphate buffer solution (0.2 mol/L, pH 7.4). After centrifugation (5,000 × g) at 4 °C for 20 min, the supernatants were stored at 4 °C for further biochemical analysis. The hepatic activities of CYP2E1, ADH, ALDH, GSH-Px, SOD, CAT and T-AOC, as well as the contents of TC, TG, MDA and LPO were analysed using diagnostic kits (spectrophotometric method) according to the instructions. In addition, the levels of TNF-α, IL-6, IL-1β, COX-2, NO and iNOS in the liver homogenate were measured using enzyme-linked immunosorbent assay (ELISA) diagnostic kits. (The main steps of TNF-α are as follows: 1. Standard, sample diluent; 2. Add the standard and sample diluent, and incubate for 30 min at 37 °C; 3. Wash 5 times, add HRP-conjugate reagent, and incubate for 30 min at 37 °C; 4. Wash 5 times, add chromogen solutions A and B, and incubate for 10 min at 37 °C; 5. Add the stop solution; 6. Read the absorbance at 450 nm within 15 min; 7. Calculate: Assay range: 25 ng/L-800 ng/L, r^2^ > 0.995; Sensitivity <5%; All of the inter- and intra-assay coefficients of variation are <10%. The other steps of the ELISA kits are similar to those for TNF-α. Additionally, the concentration of proteins was determined by a Coomassie Brilliant Blue G-250-based assay^54^. Moreover, the samples (ten mice in per group) were merged in one (the same group is incorporated into a sample tube) and measured triplely in order to calculate the SD accurately.

Fresh livers were immediately fixed in 10% buffered formalin (pH 7.4) and embedded in paraffin. After cutting (4–5 μm thickness) and staining with haematoxylin-eosin, the sections were examined under a microscope for evaluating the morphological pathological changes (×600 magnification)^1,33^. Our histological scores were assessed using Ishak’s system^55^; the scoring systems were referenced by Chen *et al*.^56^ and by Cima *et al*.^57^ and are based on a sum of three parameters: inflammation grade, cell infiltration and tissue disruption. In the scoring system, H&E staining in the livers was scored using a scale of 0 to 4 (0 = no inflammation grade, cell infiltration and tissue disruption; 1 = 0–25% inflammation grade, cell infiltration and tissue disruption; 2 = 25–50% inflammation grade, cell infiltration and tissue disruption; 3 = 50–75% inflammation grade, cell infiltration and tissue disruption; and 4 = 75–100% inflammation grade, cell infiltration and tissue disruption). Each tissue was evaluated for the sum of these three parameters and assessed by the degree of liver injury using a qualitative score that ranged from 0 to 4. A score of 0 was categorized as no damage, scores between 1 and 2 were categorized as light injury, and scores of 3 and 4 were categorized as serious injury.

### Acute toxicity experiment

The acute toxicity experiment was processed using the method reported by Zhang *et al*.^29^. Twenty Kunming strain mice (male, 20 ± 2 g, 8-week-old) were obtained from Taibang Biologic Products Limited Company (Tai’an, China) and were randomly divided into two groups, with ten mice in each group. The mice in the control group received saline solution, while the mice in the experimental groups (EPS) orally received EPS at a dose of 4,000 mg/kg, and all the mice were fed by intragastric administration (i.g.). The mice were observed continuously for their appearance parameters and for any mortality or behavioural changes (respiratory distress, abnormal locomotion, or catalepsy) during the entire feeding period (14 consecutive days)^39^.

### Statistical analysis

All the data are expressed as the mean ± S.D. (standard deviation). The ANOVA was performed using SPSS 19.0 software (International Business Machines Corporation (IBM Corporation, USA). After verifying the data followed the normal distribution, and then we analysis the analysis of variance (ANOVA). Significant differences between experimental groups were determined using a one-way ANOVA followed by Duncan’s post-hoc test, and *P* < 0.05 was considered a statistically significant difference. Any groups with the same letter are not statistically different from each other^58^.

### Compliance with Ethical Standards

The experiments were performed as approved by the Institutional Animal Care and Use Committee of Shandong Agricultural University, and in accordance with the Animals (Scientific Procedures) Act 1986 (amended 2013).

### Ethics statement

All experiments were performed in accordance with the guidelines and regulations of the ethics committee of the Shandong Agricultural University and the Animals (Scientific Procedures) Act of 1986 (amended in 2013).

All experimental protocols were submitted to and approved by the ethics committee of Shandong Agricultural University in accordance with the Animals (Scientific Procedures) Act of 1986 (amended in 2013).

## Conclusions

The present structural analysis revealed the existence of an α-glycosidic bond in the EPS, which was found to be a heteropolysaccharide by GC and FTIR, and we observed the rough surface of the EPS *via* the ultrastructures. In addition, we concluded that EPS showed potential hepatoprotective effects against the alcohol-induced liver injury mainly by improving the antioxidative status and enhancing the effects of anti-inflammatory. These results might provide a theoretical basis for EPS application in natural and functional foods for the prevention and alleviation of ALD and the complications.
